# Stem cells in human breast milk

**DOI:** 10.1007/s13577-019-00251-7

**Published:** 2019-04-10

**Authors:** Natalia Ninkina, Michail S. Kukharsky, Maria V. Hewitt, Ekaterina A. Lysikova, Larissa N. Skuratovska, Alexey V. Deykin, Vladimir L. Buchman

**Affiliations:** 1 Institute of Physiology Active Compounds, Russian Academy of Sciences, 1 Severnyj Proezd, Chernogolovka, Russian Federation; 2 Cardiff University, Life Sciences Building, Museum Avenue, Cardiff, Wales CF10 3AX UK; 3 Pirogov Russian National Research Medical University, Ostrovitianova str 1, Moscow, Russian Federation; 4 The Institute of General Pathology and Pathophysiology, 8 Baltiyskaya st., Moscow, 125315 Russian Federation; 5 Institute of Gene Biology, Russian Academy of Sciences, Vavilova str., 34/5, Moscow, 19334 Russian Federation

**Keywords:** Breast milk, Stem cells, Source of human stem cells, Regenerative medicine

## Abstract

Recent studies have demonstrated that breast milk contains a population of cells displaying many of the properties typical of stem cells. This review outlines progress made in this newly emerging field of stem cell biology and provides an analysis of the available data on purification, propagation and differentiation of certain types of progenitor cells from breast milk. The possible fates of breast milk cells, including microchimerism caused by their transmission to the distant organs of the infant, are also discussed. Unique properties of breast milk-derived stem cells, such as their unusually low tumorigenic potential and their negligible ability to form teratomas, are highlighted as obvious advantages for using these cells in regenerative therapy.

## Introduction

Breast-feeding is an indispensable post-parturition stage in the process of mammalian reproduction that is crucial for neonatal survival during critical periods of their postnatal development. Each mammalian species has a unique composition of milk that has developed during evolution to fulfill the requirements of their newborns and infants. In humans, the natural reproduction process includes a long period when all nutrients and other chemicals required for successful physical and intellectual development of a child come from milk provided by a lactating mother, emphasizing the importance of this biological product. For this reason, human breast milk has been intensively studied for many years [1, 2].

## Not just nutrition supply

It is now well known that in addition to specially balanced essential nutrients, breast milk contains functionally distinct bioactive components that are involved in remodeling of the immune system of neonates and infants, and alter their susceptibility to different types of infection [3].

Recent studies have revealed the complex supplementation mechanisms that involve a direct supply of mother’s mature immune cells as well as trafficking of other cell types supporting the development and functional regulation of both the innate and adaptive immune systems of infants [4–8]. It has been shown that certain types of cells present in breast milk are able to pass through the infant’s gastrointestinal tract and populate distant sites such as the spleen, liver and lymph nodes [9, 10]. Moreover, communication between breast milk components and their natural host, the infant, which create a symbiotic commensal relationship, have allowed some researchers to suggest that the breast milk is a live system and could even be considered an organ [11–13].

One of the most remarkable recent findings is the demonstration that infections or other changes in the status of the nursed infant trigger a rapid response in secretion, composition and cell contents of consumed breast milk. The exact mechanisms of this sophisticated feedback that support the dynamic relationship between infant’s homeostasis and efficient fulfilling of its immediate needs remain unknown [14–16]. The complexity of symbiotic interaction extends beyond the eukaryotic cells of mother’s origin and include a diverse microbiome that is presumed to colonize the infant gastrointestinal tract and, therefore, is a continuous source of beneficial bacteria such as *Bifidobacterium breve, B. adolescentis, B. longum, B. bifidum, B. dentium* and others [17–19].

Although study of probiotic bacteria in human milk is a very recent and clearly underdeveloped field of research, there is already experimental evidence that selected bacteria from the gastrointestinal microbiota of nursing females are able to access the mammary gland via the entero-mammary pathway. This transduction occurs by dendritic cells and CD18 + cells carrying nonpathogenic bacteria from the gut lumen to the lactating mammary gland [20]. It came as no surprise that the infant gut becomes actively colonized by the breast milk-supplied bacteria, which is ensured by the high content and variety of probiotic cells that on average could comprise 10^7^–10^8^ when around 800 ml of milk is consumed daily [12, 21]. This has allowed researchers to suggest that human breast milk satisfies the criteria for consideration as a probiotic food [22].

Breast milk is also a potential source of some previously unrecognized biologically active entities. One recent and very exciting finding is the demonstration that the exosomes purified from breast milk are able to promote intestinal epithelial cell growth in infants even when they are formula feeding [23]. The stimulating effect of breast milk on the growth and proliferation of enteroids generated from neonatal mice or premature human small intestine have also been shown in in vitro experiments [24]. This research further substantiates previous suggestions that breast milk could be used for therapeutic purposes in combination with conventional drug therapy [2, 25]. Taken together the results of these recent studies has substantially broadened our view of the function of human breast milk and stimulated further research utilizing new approaches and advanced modern methods.

## Progenitor cells of breast milk

New methods for the identification and separation of cell suspensions, such as multicolor flow cytometry, allow for the accurate assessment and quantification of the cell composition of biological fluids. Implementation of these methods has already significantly advanced our current knowledge about various cell populations present in breast milk. Cells of eukaryotic origin (i.e., excluding probiotic bacteria) found in breast milk can be pooled in to two major groups: blood-derived and breast-derived cells, and in both these pools small groups of progenitor or stem cells have been identified [26–29].

Not surprisingly, the largest proportion of total cell counts in breast milk is CK18^+^ luminal epithelial cells and beta-casein-positive lactocytes that synthesize milk proteins. In human milk produced by healthy nursing females feeding healthy infants luminal and myoepithelial cells together could constitute up to 98% of all cells [30]. However, the epithelial component of breast milk includes not only mature epithelial cells, but also their precursors and stem cells [30]. One of the most important and still not fully addressed questions is the identity of the source and origin of multipotent cells found in breast milk. The mammary gland employs a sophisticated machinery for converting the resting non-lactating mammary gland into a milk-secretory organ, which requires substantial expansion and cellular differentiation from the original source of progenitor cells [31–34]. Normally these stem cells remain in quiescent niches before they start asymmetric division and undergo their ductal-alveolar morphogenesis during pregnancy and lactation. Activation of certain intracellular pathways, for example the Wnt-signaling pathway, that is associated with continued morphogenesis, supports the high rate of surviving and expansion of these cells in culture [35].

The committed stem cell progeny are seen as an important source of human stem cells for therapeutic purposes [36–38]. These cells could also be advantageous for cancer research, particularly for revealing the role of proliferation-responsive cell populations in tumorigenesis, when they escape the control mechanisms that hold them in quiescence in the resting mammary gland [39, 40]. Cregan et al. have studied cultured cells from breast milk and provided the first evidence that some of these cells exhibit the properties of stem cells [26]. A substantial proportion of cells in cultures established from donor milk were positively stained for cytokeratin 5 (CK5^+^), a mammary stem cell marker. In the lactating mammary gland, CK5^+^ cells usually present in the alveoli and ducts of the epithelium and most probably they represent the source of CK5^+^ cells in cultures obtained from donor milk. However, the source of these cells and their possible role in milk is still enigmatic [41].

Other cells with characteristics typical for stem cells were also found in cultures established from cells present in breast milk. These include cells expressing α6 integrin (CD49f), a mammary stem cell marker, and an epithelial progenitor marker p63 [28, 42, 43]. Systematic in vitro research provided by Thomas et al. confirmed that a subpopulation of cells cultured from breast milk not only express stem cell markers but also exhibit the major features of multipotency. These cells are capable of renewing themselves, and under certain conditions are able to undergo differentiation towards at least two types of epithelial lineages that give origin to either milk proteins-producing CK18^+^ luminal cells, or CK14^+^ myoepithelial cells [28, 42].

These groundbreaking reports have boosted further research on the cellular components of breast milk. Remarkably, all the main findings reported in the original publications have been successfully confirmed using colony-forming assays carried out by other laboratories and our own unpublished data [12, 27]. Moreover, in culture, a subpopulation of breast milk-derived stem cells display very high multilineage potential, resembling those typical for human embryonic stem cells, hESCs [29]. This subpopulation has been designated as “hBSCs” (for human breast milk stem cells), however, it would probably be more appropriate to use the abbreviation “hBmSCs” to avoid potential confusion with the breast stem cells originated from tissue biopsy of the mammary gland.

So far, no differentiation techniques specific for cultivated milk cells into various cell lineages have been developed but methods and factors commonly used for differentiation of progenitor cells of other origin linage have been successfully employed. Sani et al. [44] cultivated purified human breast milk-derived stem cells and achieved their differentiation toward different lineages, again suggesting the presence of a pool of pluripotent cells in these cultures. A large proportion of these cells expressed the mesenchymal stem cell markers: CD44, CD90, CD271, and CD146. A smaller subpopulation of these hBmSCs also expressed the embryonic stem cell markers: Oct4, Sox2, TRA 60-1, Nanog but not SSEA1 or SSEA 4. Another population comprised of cells that expressed cytokeratin 18, a marker for luminal mammary epithelial cells, were capable of differentiating into adipocytes and osteoblasts. Moreover, under certain conditions formation of neurospheres that were stained positively for nestin and some neuron and glial-specific markers were also observed [44, 45].

In another study, the hBmSCs were found positively labeled with the stage-specific embryonic antigen 4 marker (SSEA4) as well as TRA-1-60 and TRA-1-81 antigens, both markers for undifferentiated pluripotent human stem cells. Furthermore, the ability of cultured hBmSCs to differentiate under certain conditions towards different epithelial lineages (including myoepithelial cells and lactocytes), and to form mini-mammary glands secreting milk proteins has been demonstrated [13]. Perhaps more importantly, hBmSCs appeared to be truly multipotent, since they were able to undergo the mesodermal route and differentiate into osteoblasts-like cells, chondrocytes, adipocytes and cardiomyocytes, and were also able to differentiate into cells of endodermal origin, such as pancreatic beta-like cells producing insulin and hepatocyte-like cells producing albumin. Finally, hBmSCs were additionally shown to differentiate into various cells of ectodermal origin including glia-like and neuron-like cells that express neuronal-specific markers such as βIII-tubulin [27, 29, 46]. Further analysis of expression patterns for genes, that are commonly used as markers of pluripotency, confirmed their activation in hBmSCs [47]. The levels of OCT4 (POU class 5 homeobox1), SOX2 (sex determining region Y-box 2), NANOG (nanog homeobox) and KLF4 (Kruppel-like factor 4) mRNAs were significantly increased. Immunohistochemical staining has confirmed the presence of the encoded proteins. Interestingly, the levels of expression of these genes in the fraction of freshly isolated cells from the milk samples varied between 17 donors with the highest level detected for an individual who was concurrently breastfeeding and pregnant. This is consistent with the expansion of mammary gland tissue and consequent increase of the mammary stem cell niche during pregnancy [13, 29].

The presence of a novel stem cell population with multilineage differentiation potential has also been recently identified in bovine milk. This cells isolated and characterized by Pipino et al. were able to grow as a plastic-adherent culture and differentiate into osteogenic, chondrogenic, and adipogenic lineages [48]. This heterogeneous population of epithelial-like and spindle-shaped bovine milk stem cells (bMSCs) was positive for the typical epithelial markers E-cadherin, cytokeratin-14, cytokeratin-18, and smooth muscle actin, but a subset of purified milk cells (30–40%) displayed typical mesenchymal surface antigens CD90, CD73, and CD105. Furthermore, a similar percentage of bMSCs expressing CD90, CD73, and CD105 presented the stemness markers SOX2 and OCT4 in their nuclei.

In a recent study of cell populations that exhibit a stem cell phenotype, Briere et al. compared breast milk samples obtained from feeding mothers of preterm infants and full-term infants and found obvious differences in the proportion of stem-like cells in these two types of samples. Further confirmation of this observation has been demonstrated by showing distinct patterns of expression of stem cell-specific genetic markers in theses two samples [49]. The latter results are linked to an intriguing but completely unresolved question about a potential symbiotic relationship between a feeding mother and nursing infant: if and how this relationship underlines the complement and phenotype of hBmSCs. There is some experimental evidence that the cross-talk between the infant and the consumption of milk regulates the gene expression in breast-milk cells [50, 51].

Due to the physico-chemical properties of breast milk, purification of its cellular components with a quality sufficient for the immediate analysis by flow cytometry methods that would be sensitive enough to detect the pool of cells displaying pluripotency markers is a challenging task. The main problem is the ability to discriminate between cells and noncellular components. Keller et al. have developed an improved protocol that use a recently developed staining agents DRAQ5™ far red and SYTOX^®^ blue for flow cytometry analysis of breast cells in human milk. This novel and reliable approach allowed to identify and quantify subpopulation of CD11b^+^ monocytes as well as putative stem cells positive for cell surface markers TRA-1-81 and SSEA-4 [52]. Interestingly, for TRA-1-81^+^ putative stem cells the high variations were detected in samples without significant differences between the gestational age groups.

**Fig. 1:**
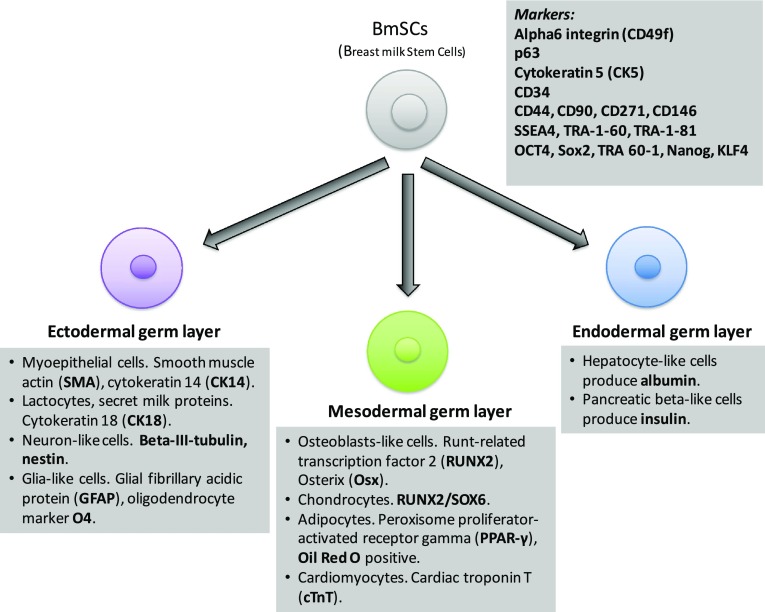
Composition of breast milk cellular component and markers of pluripotency identified in a fraction of presumptive breast milk stem cells. Available experimental evidence suggest that there is a small fraction of cells (BmSCs) displaying key properties of stem cells in breast milk amongst various types of other cells that are present. BmSCs are able to differentiate into cells of all three germ layers and the level of their pluripotency is comparable with that of human embryonic stem cells

**Fig. 2:**
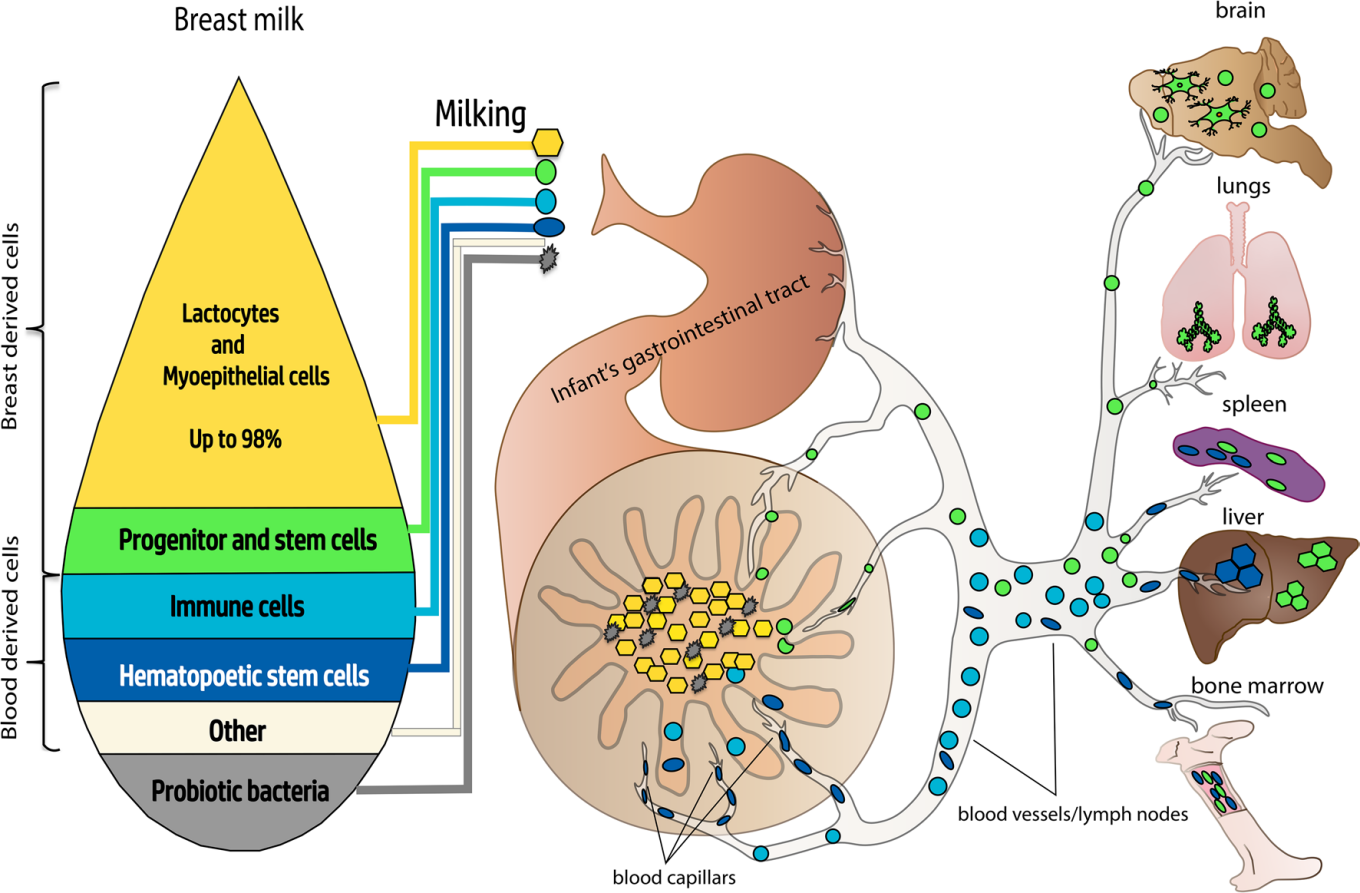
Dissemination of breast milk cells throughout the body of nursed infants. A fraction of breast milk progenitor cells is able to penetrate the wall of the gastrointestinal tract of nursed infants, entering their circulatory system and populating distant organs

Taken together, the available experimental evidence suggests that hBmSCs are able to differentiate into all three germ layers (Fig. 1) and that the level of their pluripotency is comparable with that of human embryonic stem cells. However, the amount of stem cells in the milk, their phenotype, and expression of cell markers of pluripotency could vary between milk donors [53] (Fig. 2).

So far, no published data provide strong enough experimental evidence for the origin of hBmSCs. Nevertheless, Hassiotou and Hartmann have recently suggested that at least some subpopulations of hBmSCs could have the same origin as hematopoietic stem cells [13]. The presence of other blood-derived cells in breast milk indirectly supports this notion. For example, the presence of CD34^+^ hematopoietic stem/progenitor cells has been demonstrated in colostrum and breast milk and it is highly probable that they originate from the maternal bloodstream [41, 54]. However, the exact mechanisms of their delivery into breast milk as well as the physiological roles of hematopoietic stem/progenitor cells in early postnatal development in infants remain unclear.

## Microchimerism caused by transmission of breast milk cells

Transmission of cells from mother to fetus and from fetus to mother via the placenta, and from feeding mothers to nursing infants via breast milk has been studied for several decades and is well documented [9, 10, 55–60]. Therefore, there is little doubt about the existence of efficient mechanisms of seeding infants with maternal cells, although their functional consequences and importance for the recipient remain elusive. There is some experimental evidence that hBmSCs can cross the wall of the gastrointestinal tract of nursed mouse pups, enter their circulatory system and reach different organs where they differentiate and become fully functional [61]. This phenomenon creates a type of microchimerism similar to that described as a result of stem cell exchange between the mother and the fetus in utero. In the latter case, seeded cells were able to survive in the chimeras for several years [55, 62–64] and it is feasible that cells which originated from breast milk might follow the same fate. Studies on breastfed rabbits have demonstrated that purified milk-derived stem cells labeled with PKH26 Red Fluorescent Cell Linker were engrafted into the offspring organs and were detected in liver, cartilage, bone and duodenum [65]. In the very recent research by Aydın et al., lactating transgenic female mice expressing green fluorescent protein (GFP^+^) were used for fostering and breastfeeding wild-type newborn pups. The maternal GFP^+^ cells in the suckling’s blood and in the brain have been identified by fluorescence-activated cell sorting, polymerase chain reaction and immunohistochemistry. Moreover, the authors have provided experimental evidence that GFP^+^ cells have differentiated into both neuronal and glial cell types in the brain after being foster fed by GFP^+^ female mice. These data strongly suggest that GFP^+^ cells were delivered to offspring by breast milk and were able to differentiate into different types of cells [66].

Research on microchimerism generated by consumed breast milk cells has firmly supported the important physiological role of this process in the efficient maturation of the infant immune system [67, 68]. For example, one possible consequence of microchimerism is an increased tolerance of the recipient to donor antigens and this evidence is indirectly supported by the improved acceptance of maternal transplants by individuals who were breastfed as infants [69, 70]. Other effects of breast milk cell-induced microchimerism cannot be excluded and, therefore, further studies are required to understand its mechanisms and physiological role.

## Potential therapeutic use of breast milk-derived cells

The discovery of hBmSCs and identification of cells differentiated from hBmSCs in various organs of recipients paved the way to the idea that breast milk is a novel promising source of transplantable stem cells for therapeutic use. An important reason why hBmSCs are included in the list of prospective sources of human pluripotent cells for use in regenerative medicine is their unusually low tumorigenic potential and the negligible ability to form teratomas [29]. Another major advantage of hBmSCs is relatively easy access and straightforward harvesting that does not involve any invasive techniques. Therefore, for some patients they represent a promising source of cells for autologous transplantation, particularly if further progress in regulated induction of milk production by dormant breast tissue and detailed characterisation of the cell composition of induced milk is achieved [71].

So far, stroke therapy seems to be the most attractive application for hBmSC transplantation. In a comprehensive report that compared various stem cell types for their potential efficacy to sequester stroke-induced neuroinflammation, feasibility as translational clinical cell sources, safety and suitability as a transplantable material, hBmSCs have been suggested as a promising source of stem cells for treatment of a stroke-associated pathology [72]. This concept for the potential therapeutic benefit of hBmSCs is largely based on results of in vitro studies where hBmSCs were cultured and differentiated in vitro followed by identification of the growth factors that they secreted [73, 74]. Of particular importance for stroke therapy is the ability of certain subpopulations of these differentiated cells to produce VEGF and HGF and a protocol for culturing breast milk-derived cells that increase production of these factors has been established [73].

Other recently developed protocols allow in vitro differentiation of hBmSCs into various types of neuronal and glial cells, including pentraxin3 producing astrocytes [29, 75]. Since pentraxin 3 can promote blood brain barrier (BBB) integrity, these hBmSC-derived astrocytes may have the capacity to encourage suppression of peripheral immune invasion after ischemic stroke. Another potentially beneficial effect of transplanted hBmSCs on BBB is due to their ability to differentiate into endothelial-like cells that could regulate the immune microenvironment in the brain regions affected by stroke and might contribute to local restoration of the BBB [72].

The current major limitation for clinical applicability of breast-milk-derived stem cells for treating stroke is the lack of knowledge about their therapeutic potential in vivo, particularly about their restorative effects, efficacy and safety [76]. For the comprehensive use of breast milk-derived cells in regenerative therapy, there is an immediate need for characterizing properties of the milk cellular component after induced lactation. There is currently no data on the cell composition and pluripotent characteristics of induced breast milk, in which, production has not been the consequence of child birth but caused by hormonal stimulation, for example in case of adoptive female mother or transgendered male-genotype mother, although the methods of induced lactation are well established [77–79]. Therefore, studies in animal models are still required before hBmSCs can be firmly considered for therapeutic intervention for stroke and potentially, other human diseases. These studies should be focused on revealing and characterizing the pattern of cell composition and the potential teratogenic properties of cells present in induced breast milk. Another important line of future research is the development of new methods for fine tuning the stem cell composition of induced donor milk.

In conclusion, there are more questions than answers in the story of breast milk stem cells but this field is rapidly gaining momentum and might bring surprising results and unexpected benefits.
